# Exploring the views of young women and their healthcare professionals on dietary habits and supplementation practices in adolescent pregnancy: a qualitative study

**DOI:** 10.1186/s40795-018-0254-7

**Published:** 2018-11-12

**Authors:** Rachel Rundle, Hora Soltani, Alexandra Duxbury

**Affiliations:** 1 Food and Nutrition Group, Sheffield Business School, Sheffield Hallam University, City Campus, Howard Street, Sheffield, S1 1WB UK; 2 Centre for Health and Social Care Research, Faculty of Health and Wellbeing, Sheffield Hallam University, Sheffield, UK

**Keywords:** Adolescent pregnancy, Dietary habits, Nutrition supplementation, Behaviour change

## Abstract

**Background:**

Nutrition is a modifiable factor affecting foetal growth and pregnancy outcomes. Inadequate nutrition is of particular concern in adolescent pregnancies with poor quality diet and competing demands for nutrients. The aim of this study was to explore knowledge and understanding of nutrition advice during adolescent pregnancy, and identify barriers and facilitators to dietary change and supplementation use in this vulnerable population.

**Methods:**

Semi-structured interviews were conducted with young women and key antenatal healthcare providers: midwives, family nurses and obstetricians. Doncaster, Manchester and London were chosen as sites offering different models of midwifery care alongside referral to the Family Nurse Partnership programme.

**Results:**

A total of 34 young women (adolescents aged 16–19 years) and 20 health professionals were interviewed. Young women made small changes to their dietary intake despite limited knowledge and social constraints. Supplementation use varied; the tablet format was identified by few participants as a barrier but forgetting to take them was the main reason for poor adherence. Health professionals provided nutrition information but often lack the time and resources to tailor this appropriately. Young women’s prime motivator was a desire to have a healthy baby; they wanted to understand the benefits of supplementation and dietary change in those terms.

**Conclusion:**

Pregnancy is a window of opportunity for improving nutrition but often constrained by social circumstances. Health professionals should be supported in their role to access education, training and resources which build their self-efficacy to facilitate change in this vulnerable population group beyond the routine care they provide.

**Electronic supplementary material:**

The online version of this article (10.1186/s40795-018-0254-7) contains supplementary material, which is available to authorized users.

## Background

The increased risk of poor birth outcomes and adverse health consequences for mother and child are well documented for adolescent pregnancy [1–4]. Nutrition is a potentially modifiable risk factor but one that is challenging to address [5, 6] given the poor dietary patterns associated with adolescent young women, particularly those from socially disadvantaged groups, which experience higher rates of adolescent conception [7, 8]. Young women with habitually poor dietary intake are at increased risk of nutritional deficiencies when entering pregnancy. Without dietary modification or supplemental nutrition interventions during pregnancy this may decrease the likelihood of their baby achieving an adequate birth weight [1].

Addressing diet and nutrition is central to improving birth outcomes and maintaining well-being throughout pregnancy. In the UK routine antenatal care for adolescent pregnancy prompts discussion of diet and nutrition at key points within the care pathway [9–11]; this covers nutrition supplementation, food-acquired infection (foods to avoid) and the provision of “*information on the benefits of a healthy diet and practical advice on how to eat healthily throughout pregnancy... tailored to the woman’s circumstances*” [12]. In the face of complex, competing social and emotional issues associated with adolescent pregnancy [8] the time available and approaches taken to addressing these factors effectively are less well known. Therefore, whilst the majority of young women will receive some nutrition guidance during their pregnancy it is unclear how much they understand, whether they make any changes to their diet as a result and the extent to which they adhere to recommendations for nutrition supplementation. For such a nutritionally vulnerable population it is important to understand how existing practices impact on their choices and the additional support required, for both young women and their ante-natal care teams.

Therefore the objectives for this study were; (i) to explore young women’s knowledge and understanding of diet and nutrition advice during pregnancy; (ii) to identify whether they make any changes to their dietary intake and what prompts these changes; (iii) to understand young women’s use of nutrition supplements before and during pregnancy; and (iv) to explore sources of information and support regarding diet and nutrition.

## Methods

### Study design

In this multicentre explorative study, the views of key antenatal care providers and a diverse population of pregnant adolescents were sought. In the UK midwives provide the majority of antenatal care with the youngest and most vulnerable being referred for obstetric support. In some areas the Family Nurse Partnership [13], provides additional care and support to young women assigning them to a family nurse practitioner for intensive intervention until their child is 2 years of age. Therefore, views of obstetricians, midwives, and family nurse practitioners were sought alongside young women. The data collection strategy and interview guide were developed in consultation with a research user group (comprising five young women (antenatal and postnatal) one midwife and one family nurse). The user group made valuable suggestions in terms of terminology (preferring terms such as *“food you choose to eat”* rather than *“your diet”*, *“healthy eating”* rather than *“nutrition”* and *“young women”* or *“young mums”* rather than *“teens”* or *“teenagers”*) interview structure and incentives which informed recruitment and data collection.

### Settings

Doncaster, Manchester and the London boroughs of Lambeth and Southwark were chosen as research sites offering different models of service provision for pregnant young women. During the study period, Doncaster and Manchester offered community midwifery led services with one specialist midwife in young women’s pregnancies for the service, locally referred to as the “teen pregnancy midwife” by other health professionals; this is noteworthy given the user group’s preference in terminology for *“young mums”*. London offered a specialist case-loading midwifery led continuity care model where young women were assigned to one key specialist midwife for their pregnancy with support from other members of the midwifery team. At the time of study the family nurse partnership followed the national model at all three research sites [13]. Obstetricians across the three sites provided specialist support for high risk pregnancies in young women.

### Participants and recruitment

A purposive sampling strategy was employed for data collection. Selection criteria for young women were based on age and pregnancy status; 16–19 years whilst pregnant, currently pregnant or had given birth less than 6 months previous. Young women were provided with an information sheet and referred to the study by their midwife or family nurse practitioner based on our selection criteria; these details were confirmed whilst written consent was obtained. All young women were offered a £20 high street voucher in appreciation of their time. Health professionals were also purposefully sampled to include obstetricians and family nurse practitioners with the majority being midwives (specialists in young women, community and hospital). Interviews were conducted in quiet spaces within the ante-natal setting, in community venues or in young women’s homes.

### Data collection

Semi-structured interviews were designed to explore diet, supplementation use and sources of information and support during pregnancy. Interviews were conducted independently by two researchers and lasted between 10 and 42 min (mean time 25 min). The researchers were experienced in the collection of interview data and had prior experience working with young women from more socially disadvantaged backgrounds, both having previously worked in the National Health Service in community outreach programmes. A visual interview guide was used to keep conversations on topic and act as a focal point e.g. to limit distraction from mobile phones (see Additional file 1 Young Women’s Interview Guide). The guide included photos of routinely used information sources, different types of nutrition supplements and tablet formats to demonstrate variations in tablet size; the same guide with minor changes to wording was used with the health professionals. During the interviews with young women the researchers sought to put them at ease and establish a rapport, reassuring them that their individual experiences were important and that there were no “right or wrong” answers. The majority of young women talked freely but some of the shorter interviews (<15mins duration) were a result of young women giving very brief responses. Young women that were pregnant at the time of interview were asked to focus on their experiences up to that point and postnatal young women were asked to think back to their pregnancy. Health professionals were asked to focus on their experiences and perceptions working with adolescent young women in their current role; this was particularly relevant for family nurse practitioners that had previously worked as midwives or health visitors. Interviews were audio recorded and professionally transcribed verbatim. The initial audio recordings and transcriptions were reviewed by both researchers for consistency and completeness.

### Data analysis

The data was analysed thematically using the approach advocated by Braun and Clarke [14]. Through careful reading, data coding, categorisation of codes and constant comparative analysis, similarities and differences were explored in order to develop the overarching themes and subthemes. The main data analysis was managed by two researchers independently and verified by a third researcher (not one of the interviewers) to reduce the potential for bias and “sense check” during theme/sub-theme development thus reducing ambiguity of meaning. It was not the purpose of the analysis to compare different models of service provision at each research site rather highlight the aspects of care that were barriers to or facilitated young women to make healthier food choices and take their supplements.

## Results

Fifty four in-depth interviews were conducted across the three study sites; young women (*n* = 34) and health professionals (*n* = 20). The characteristics for young women are presented in Table 1. All health professionals were involved in some aspect of antenatal care. Midwives made up the greatest proportion of the sample (*n* = 12) followed by family nurse practitioners (*n* = 5) and obstetricians (*n* = 3); of the midwife interviewed five had specialist adolescent pregnancy roles, two as *“teen pregnancy”* specialist for their locality (Doncaster and Manchester) and three with adolescent pregnancy caseloads (London).

**Table 1:** Participant characteristics for young women (aged 16–19 years) recruited across the three interview sites (Doncaster, Manchester and London)

Variable	n (%)	mean
Age (years)		17.62 years
16	4 (12)	
17	13 (38)	
18	9 (26)	
19	8 (24)	
First pregnancy	33	
Second pregnancy	1	
Stage of pregnancy at interview		
Pregnant	24 (70)	
Gestational age (weeks)		28 weeks
Postnatal	10 (30)	
Weeks		14 weeks
Living situation		
Alone, own home (rented)	3 (8.5)	
With partner, own home (rented)	10 (30)	
With family, at home	12 (17)	
With partner and family, at home	6 (17)	
Shared house, hostel accommodation	3 (8.5)	

### Theme 1: A time for dietary change amidst confusion

(See Additional file 2: Table S2 of results for “Diet” theme and sub-themes).

Pregnancy was acknowledged by the majority of interviewees as a time for dietary change yet the opportunity to make healthier dietary choices was often hampered by misconceptions and entrenched food preferences. A “*typical teenage diet*” was how many health professionals referred to young women’s food choices with lack of understanding regarding the impact of dietary intake affecting their motivation to change. Midwives and family nurse practitioners often spoke about developing young women’s *“connection with their baby”* as a means of initiating dietary change. Where young women made this connection, “*All the way through, like, I just eat healthier because then your baby will come out healthie*r”, more regular eating patterns and healthier food choices were reported. Conversely misaligned beliefs about what they ate and their baby’s development were a barrier to positive change. Young women reported restricting food intake, motivated by a desire for a *“smaller baby”* in the belief that it would ease labour; health professionals highlighted the importance of dispelling these misconceptions.

Food safety advice often prompted dietary changes but young women could recall few specifics when asked; they admitted to feeling confused which led them to exclude certain foods such as tinned tuna, eggs, nuts and cheese, in particular processed soft cheeses,. Midwives expressed concern that some of these foods (e.g. cheese, eggs and tuna) could provide potentially cheap and useful sources of nutrients (e.g. calcium and protein). Yet despite their confusion young women’s motivation was primarily to protect their baby; they believed consuming these foods may be harmful, even though they did not understand why, and they erred on the side of caution by excluding them completely from their diet.

Health professionals often described young women’s dietary choices as being of poor nutritional quality, expressing concern about missed meals and long periods of time between eating. Where young women reported making healthier choices these were not in the absence of junk food, crisps and confectionary but small positive changes to an overall poor diet; for example, one portion of fruit per day where none had been consumed prior to pregnancy, swapping carbonated sugary drinks for fruit juice, milk or water or eating breakfast.

Young women’s living arrangements, access to cooking facilities and financial constraints all influenced food choice. Cooking was not a regular feature of their day and they relied on others cooking for them (mother, grandmother or partner’s family). Some health professionals felt that living with family meant young women had a more balanced and nutritious diet but others felt this was not the case where the habitual family diet was reliant on take-away and convenience foods. Young women living alone or in temporary accommodation were least likely to cook for themselves. A few young women, particularly those with a supportive partner, spoke about taking more interest in cooking and trying new foods during pregnancy.

Young women’s use of the Healthy Start voucher scheme [15] which provides monetary assistance for purchasing fruit, vegetables and milk during pregnancy was also explored during the interviews. They reported using the vouchers for fruit and milk or as contribution towards the household food shopping. The application process for the vouchers was a barrier and decreased motivation for some young women. One family nurse highlighted misuse of the vouchers as an issue in her locality although this was attributed to small local retailers. Whilst this was corroborated by some young women they were not concerned when they were able to exchange the vouchers for ineligible foods such as bread. Health professionals also felt that supporting development of shopping and cooking skills alongside use of Healthy Start vouchers could improve confidence to make small dietary changes in young women that in many cases are fending for themselves for the first time.

### Theme 2: Erratic adherence to supplementation despite uncertainty

(See Additional file 3: Table S3 of results for “Supplementation” theme and sub-themes).

Use of nutritional supplements during pregnancy varied amongst the young women and was often challenging to explore when they were confused about what they were taking and why. Some young women denied taking any pregnancy supplements, associating them with *“medicine”* which they had been told to avoid. Often as interviews progressed they would talk about *“starter vits”* or *“folic something”* and by sensitive exploration the interviewer was able to elicit what supplements they were taking (if any), when and why.

Whilst the majority of young women reported taking a nutritional supplement at some stage of their pregnancy they often took them erratically and started later, towards the end of their first trimester. Later presentation of pregnancies was commonly reported by midwives and they were concerned the delayed initiation of folic acid supplementation resulted in the critical first trimester being missed. They felt being able to provide folic acid or Healthy Start vitamins directly to young women at first contact (8–10 weeks), rather than a prescription or application for Healthy Start, would provide an opportunity to reinforce the importance of taking supplements. One obstetrician expressed a desire to be more proactive in their approach to identifying those at risk of nutritional deficiencies earlier in pregnancy.

Young women were most knowledgeable of the recommendation for folic acid; prior to their initial contact with a midwife or GP, a supportive partner, family or friend was more likely to prompt taking folic acid than other types of supplement. Some young women did not realise the Healthy Start vitamins contained folic acid and were safe to use throughout pregnancy. They considered them *“not safe after 12 weeks”* and were concerned about *“overdosing”* or *“harming my baby”* if they continued. Young women described iron as being important for *“blood or brain”* but had poorer knowledge of vitamin D. Midwives described the routine haemoglobin test at 20 weeks as an opportunity for discussing dietary modification and supplementation where necessary. The use of multi-vitamins and fish oil supplements was less common but these were sometimes purchased by a well-meaning partner or relative. Young women considered them expensive but the premium packaging and marketing alongside these products made them wonder whether they were better than folic acid or Healthy Start vitamins alone. Some midwives also reported recommending multi-vitamin supplements in circumstances where they were particularly concerned about poor habitual dietary intake.

Health professionals frequently reported to young women’s reluctance to take supplements in the tablet format but the most common reason for young women not taking them was simply forgetting. Some young women reported feeling nauseous when the tablet size was too large, (more commonly with folic acid/iron combinations and vitamin D) or when taking them on an empty stomach or with sugary carbonated beverages. Young women that were motivated to take their supplements set their own prompts such as putting the tablets in a memorable place (e.g. by their toothbrush, kettle or in their purse), setting an alarm on their mobile phone or enrolling a willing partner or mother to remind them. Midwives used results from routine blood tests to prompt young women to take their supplements and tried to incentivise them by promoting baby’s healthy development and fewer pregnancy complications.

Alternative supplement formats were only made by two young women; these were for fortified products such as “*healthy chocolate*” or fruit smoothies. Few health professionals suggested alternatives, usually a fortified biscuit, but they acknowledged that it would be challenging to find an acceptable alternative with young women’s varied food preferences. It was also noted that where healthcare trusts experienced difficulties with the supply and distribution of Healthy Start vitamins this would not be eased by a more costly and bulkier product to provide the necessary supplementation. In addition, it would be counter-intuitive to promote “*healthy chocolate*” or “*healthy biscuits*” when these are the foods young women try to reduce or substitute in an attempt to make small positive dietary changes.

### Theme 3: Seeking relevant and reliable information and support

(See Additional file 4: Table S4 of results for “Information & Support” theme and sub-themes).

Midwives and family nurses felt that providing information and support on diet and nutrition was part of their role. Obstetricians described supporting the most vulnerable young women but assumed discussions about diet and supplementation would be provided by the midwifery teams. Partner, family and friends were highlighted as influential sources but the accuracy and consistency of their advice was questioned by both young women and health professionals. Midwives and family nurses recognised the trust that develops between themselves and the young women they care for. Yet the challenge of tailoring information to meet young women’s needs, given the pressured environment in which they work was acknowledged by all health professionals. The majority of midwives were confident providing routine information but admitted that time constraints and other priorities meant conversations were brief and not always relevant to young women’s situation. Midwives were frustrated they could not do more, particularly specialist *“teen pregnancy”* midwives that were the only person in that role for a particular locality. Health professionals identified a lack of confidence and practical skills as key barriers for young women putting dietary advice into practice. Family nurses acknowledged their unique position in having more time to spend with young women, which enabled them to tailor information and provide practical and emotional support. Family nurses spoke about their training and specially developed programme resources designed to help young women make the connection with their baby and offer a more holistic view of diet, supplementation and pregnancy.

Leaflets and booklets were routinely provided during antenatal appointments but there was mixed response to these. Some young women valued a resource they could keep and refer to after the appointment whilst others expressed apathy unless they had been developed specifically for adolescent pregnancy (e.g. Tommy’s Young Women’s Guide to Pregnancy). Midwives acknowledged their limitations and some reported to using them sparingly. However, young women’s experience of receiving leaflets with little opportunity for discussion often left them feeling overwhelmed and confused. Alternative sources of information were explored with websites, apps for mobile devices, You Tube (video) clips and online discussion forums being the most accessible. Social media was frequently used by young women for seeking information and sharing their experiences. They valued the experiences of other young women, particularly when it came to practical tips and ideas for making changes, which were seen as more relevant to their needs. Health professionals acknowledged young women’s prolific use of such social media platforms but were wary of user-generated content. Young women expressed concern about the reliability and accuracy of some online information. Midwives used online resources as part of their routine care but whilst most were familiar with branded sites such as NHS Choices [16] they were less familiar with other sources. Some midwives reported using You Tube videos, to deliver key messages in a more engaging manner.

Young women’s understanding and interpretation of the information received during pregnancy had the potential to influence the changes they made. Making the connection with their baby appeared to influence young women’s motivation to change; for some this was a natural result of being pregnant but others struggled to make the connection due to lack of physical changes, heightened concern with their own bodies changing (weight gain) or other competing priorities. Some health professionals felt that over and above providing information about healthy eating and supplements their responsibility lay in helping young women foster their connection with baby by developing an awareness of the impact their choices on their baby’s health.

## Discussion

This multi-centre qualitative study provides an in depth insight into young women’s dietary behaviours and supplementation use during pregnancy. The findings from our study illustrate that pregnancy is a window of opportunity when young women are willing to change albeit constrained by social circumstance and lack of intrinsic motivation. Evidence of what works to improve the nutritional status for this vulnerable population group is limited but through our analysis we have attempted to identify existing good practice, highlight the challenges and propose mechanisms for facilitating change; both behaviour change for young women and changes in practice for healthcare teams and the support they provide.

### Making the connection with baby and enabling dietary change

Studies have shown that pregnancy is a time when women are motivated to make small changes to health behaviours, but that changes to dietary patterns are limited and the extent to which they impact on nutritional status unknown [17, 18]. A recent survey of dietary beliefs and behaviours of pregnant adolescents [19] found that whilst participants recognised the importance of dietary intake this knowledge did not always translate into behaviour. Yet our study recorded accounts of dietary change, albeit small, that were made despite lack of understanding, social support and financial constraints. Our young women expressed a desire to do the best for their baby, attempted and appeared more willing to change than health professionals gave them credit for. The extent to which our young women’s dietary changes impacted on their nutritional status is unknown but reinforcing small steps in the right direction and increasing young women’s self-efficacy to change would be a valuable approach for health professionals.

Studies have acknowledged midwives central role in the provision of nutrition education and counselling during pregnancy [20] and their role in motivating and effecting dietary change [21]. However, we found that whilst the majority of health professionals provided nutrition information the extent to which this was tailored to young women’s needs and enabled change varied considerably. The barriers to providing adequate, timely and tailored nutrition support are acknowledged by ours and other studies; a directive, risk-reduction approach to providing dietary information [22] plus lack of time and appropriate resources [23] impact on women’s experiences and motivation. Information overload without opportunity for discussion can result in young women feeling confused and overwhelmed. Health professional’s nutrition knowledge and self-efficacy in tailoring information and support were also identified as important in our study. The need for improved post registration nutrition training and skills development for midwives [20, 24] plus a better understanding of how this can impact on practice and pregnancy outcomes [25, 26] has been identified. Understanding of specific techniques and approaches with regards to dietary behaviour change, particularly within disadvantaged and vulnerable population groups is still limited. Supporting health professionals to effectively direct the nutrition education and support they provide is essential to enable sustained dietary changes. Therefore, drawing on wider understanding of how different behaviour change techniques can be employed to effect dietary change [27, 28] alongside improved nutrition education would be worthy of exploration.

### Pregnancy resources as an adjunct to care and in an accessible format

It was acknowledged in our study that there already exists a wealth of resources to support healthy choices in pregnancy but these are not always tailored to young women’s needs or available in accessible formats. Information that is representative of young women’s experiences and acts to complement health professionals in their role is needed. We found that leaflets were routinely used to provide information on diet and supplementation with mixed response from young women; whilst some young women welcomed a resource they could take home and read at a later time, often, especially where these were provided without opportunity for discussion, young women were left feeling overwhelmed and confused, which mirrors findings from other studies [21, 23, 29]. Studies have shown that where time constraints and other pressures result in leaflets being provided without opportunity for discussion this reinforces hierarchical relationships [30] which could limit young women’s motivation and self-efficacy to change. Supporting health professionals in their role by utilising new communication networks, such as web-based and mobile technology, could be a means of easing time constraints by increasing accessibility of tailored information beyond routine antenatal contact.

The use of the internet, particularly by young women, is widely accepted as a means of seeking information during pregnancy [31, 32] and has been shown to be effective as a means of providing emotional support for isolated young mothers [33]. Similar to the health professionals in our study other research has found that health professionals are more likely to express reservations and lack of skills in using technologies effectively [32]. Drawing on existing evidence of how adolescents use social media to access health information [34] and how health literacy skills can impact on accessibility and use of online information [35] are all worthy of further exploration within pregnant adolescents. The potential for digital and social media opportunities to support dietary behaviour change have been acknowledged [36] although specific approaches and methodologies are still developing.

### Supplementation – Understanding benefits, barriers and access to pregnancy vitamins

Poor adherence to recommendations for pregnancy supplementation is not unique to adolescents and studies have highlighted women’s negative attitudes and ambivalence [37–39]. Yet our findings show that where women understand supplementation recommendations in terms of their baby’s health and development, and identify practical strategies to prompt use, these barriers may be overcome. Our findings also echo those found in a study exploring the uptake of Healthy Start vitamins and vouchers in England [40] where health professionals experienced difficulties with the supply and access to vitamins which reduced uptake of vitamins in those eligible for the scheme. Universal provision of Healthy Start vitamins to all pregnant women has been proposed [40] but this was not considered cost effective in a recent review by NICE [41]. Other studies have found that direct provision of folic acid to women, in addition to counselling and reminder phone call, increased weekly intake of folic acid [37]. More research is needed to establish whether the mode of accessing vitamins (direct provision from health professionals rather than the existing application and assessment process) plus behavioural counselling can increase uptake and use, particularly in nutritionally vulnerable populations.

### Strengthening the evidence base for effective dietary intervention

Whilst there is evidence that intervention to improve nutrition through foods, fortified foods and nutrition counselling may have benefits in terms of birth outcome, particularly for low income and nutritionally vulnerable populations [42, 43], there is a dearth of evidence for effective interventions in pregnant young women and adolescents in particular. A recent systematic review of nutritional interventions and birth outcomes with this population group identified only five studies eligible for inclusion and only two of the five reported significant effect on birth outcome [44]; one study using zinc supplementation reducing the likelihood of low birth weight and the other increasing average birth weight with four servings of dairy each day. The challenge of implementing interventions to change dietary behaviour in a particular group has been acknowledged and recognises the need to also change the behaviour of those delivering the intervention [45]. More research is needed which combines improving knowledge, skills and self-efficacy in health professionals to facilitate behaviour change with targeted dietary outcomes based on the available evidence.

### Strengths and limitations of the study

The strength of this research is the combined exploration of young women’s and health professional’s experiences of dietary change and supplementation use in adolescent pregnancy; we have also considered their different perspectives in terms of access to information and support and their relevance to young women’s needs. We have considered how the synergies and differences between young women’s and health professional’s views can provide insights into changes in practice and where additional research is needed. In qualitative studies such as this expectations of the researchers prior to the data collection should always be acknowledged. In this instance, based on literature reviews and anecdotal evidence from health professionals, young women were assumed to have limited interest in nutrition and resistance to dietary change during pregnancy. However, the researchers approached the interviews with an open mind and a willingness to explore young women’s experiences and they found their initial pre-conceptions challenged. Adolescent pregnancies, as represented by the young women interviewed, should not be viewed as a homogenous group whose nutritional needs can be met by a simple one-size fits all intervention. The diversity of opinion and willingness to change within the young women interviewed reinforced the researcher’s belief that their experiences should be heard and better ways of supporting them, during pregnancy and beyond, should be explored.

Limitations of the research primarily concern the age and representativeness of the young women in the interview sample; the youngest (< 16 years) and potentially most vulnerable (those not accessing routine antenatal care) may be under-represented. However, the method of recruitment enabled midwives and family nurse practitioners to refer some of their most vulnerable young women to the research team and the use of high street vouchers as an incentive may have provided encouragement to participate irrespective of individual’s prior knowledge or interest in nutrition.

To ensure reliability of the methods for data collection the researchers (interviewers) worked closely together checking the questioning style and topic coverage within the interview guide. However, there will be inevitable differences in style and rapport of the interviewer with the study participants and it is acknowledged that this may have had an impact of the reliability of data collected. Reliability of the data analysis and interpretation of codes into themes and sub-themes was aided by the oversight of the third researcher to ensure the decisions made were clear and transparent. Throughout the analysis process discussions were had to ensure that the personal viewpoints and experiences of the researchers were recognised but did not unduly impact on the impartial interpretation of the findings. We have sought to represent the experiences and viewpoints of all participants in this research as truthfully as possible to ensure the validity of our findings; whilst they may not be generalisable across all adolescent pregnancies and approaches to healthcare in the ante-natal setting we believe they offer valuable insights.

## Conclusion

Supporting young women to improve dietary quality and adhere to recommendations for pregnancy supplementation is complex. The habitual diet of young women and their families, combined with a lack of knowledge, confidence, skills and encouragement contribute to entrenched behaviours that are difficult to change. Diet may be low on the list of priorities for young women and their health professionals, particularly when faced with more immediate health and social concerns. Yet, as so many of the young women in our study reported, pregnancy is a time when they are prepared, willing and motivated to change, however small those changes may be. Whilst acknowledging that young women face numerous challenges, their prime motivator is a desire to have a healthy baby; they need to understand the benefits of supplementation and how healthier dietary choices can enhance nutrition and outcomes for their baby in those terms. Health professionals should be supported in their role to access education, training and resources which build their knowledge, skills and confidence to facilitate change in this vulnerable population group beyond the routine care they provide. This is an opportunity not to be missed as the potential benefit for mother and baby are lifelong, including improved pregnancy outcome and healthier habits into childhood and beyond.

## Additional files

Additional file 1: Young Women's Interview Guide. (PDF 1208 kb)

Additional file 2: **Table S2.** Diet theme, sub-themes and coding with illustrative quotes from young women and health professionals. (DOCX 22 kb)

Additional file 3: **Table S3.** Supplementation theme, sub-themes and coding with illustrative quotes from young women and health professionals. (DOCX 20 kb)

Additional file 4: **Table S4.** Information and support theme, sub-themes and coding with illustrative quotes from young women and health professionals. (DOCX 20 kb)
