# Supplementary material for: Exploring the views of young women and their healthcare professionals on dietary habits and supplementation practices in adolescent pregnancy: a qualitative study
Source: BMC Nutr. 2018 Nov 12;4:45. doi: 10.1186/s40795-018-0254-7 (PMC7050931; doi:10.1186/s40795-018-0254-7)

Keeping Healthy during Your Pregnancy

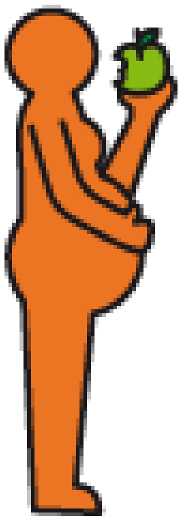

Young Women's Interview Guide
May 2013

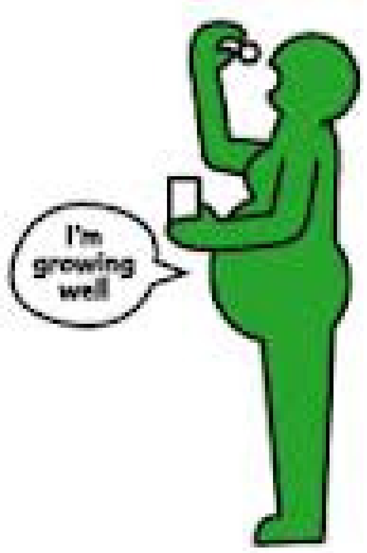

Our project...

We know that you often receive lots of information about what to eat and how to keep healthy during pregnancy.

We want to know what has been useful...

And if there's anything else we can do to make it easier for you to choose healthier food and understand how it can help you and your baby...

Your experiences....

There are no right and wrong answers...
We're just interested in what you do & why...

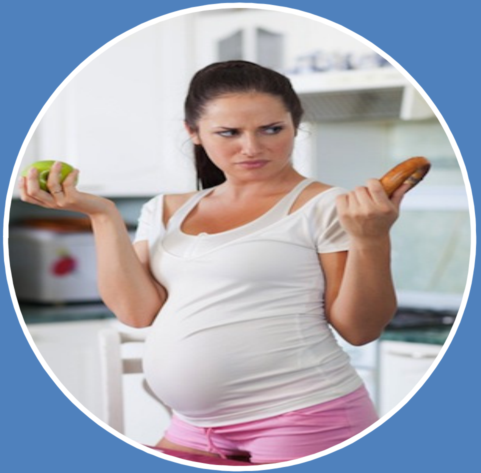

FOOD

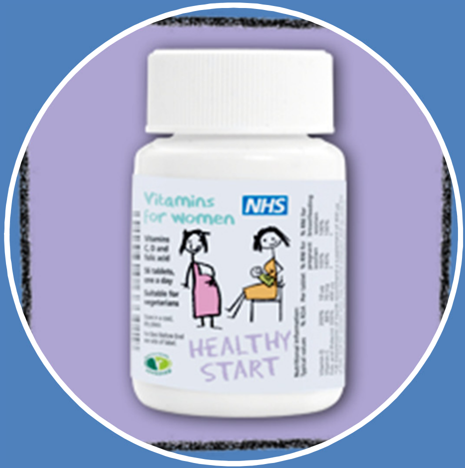

VITAMINS & HEALTHY START

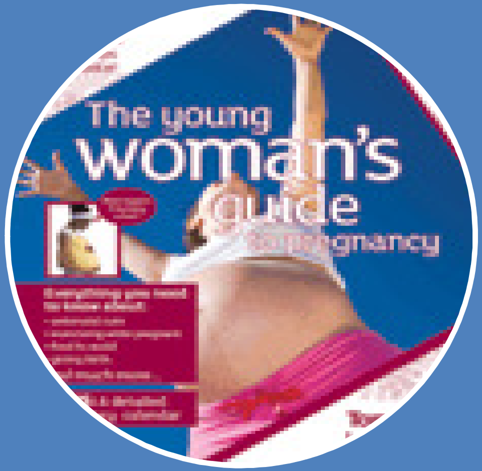

Info & Support

YOUR EXPERIENCES & IDEAS FOR FUTURE

The food you eat....

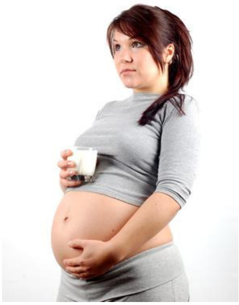

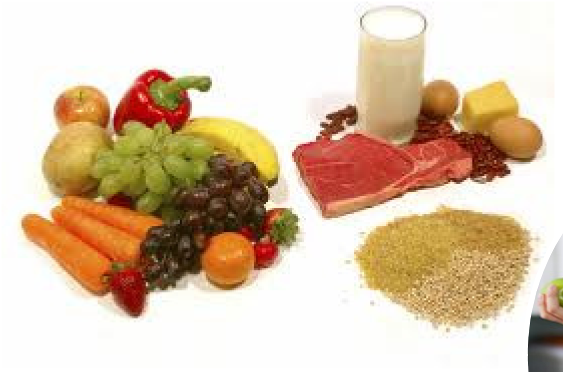

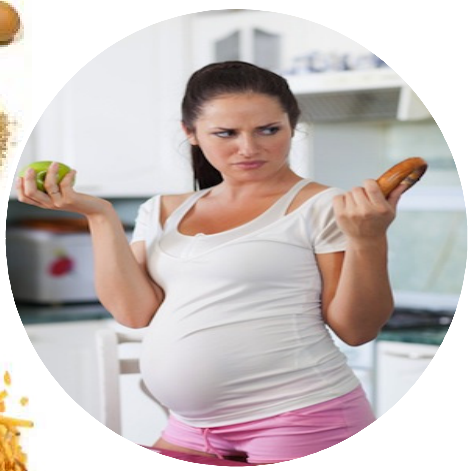

- **Meals & snacking**
- **Foods you eat/avoid**
- **Changes in pregnancy**

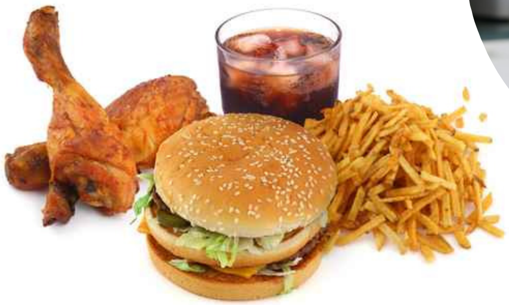

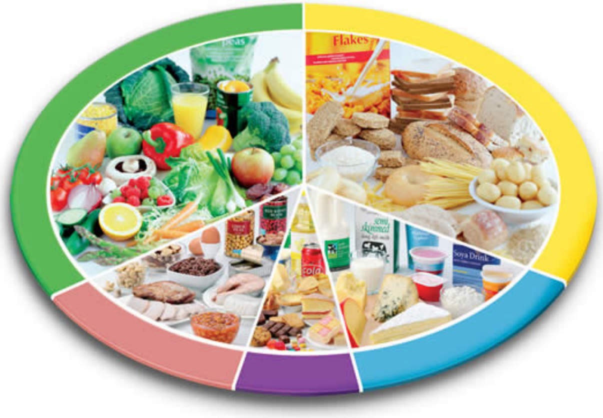

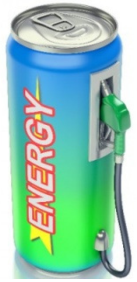

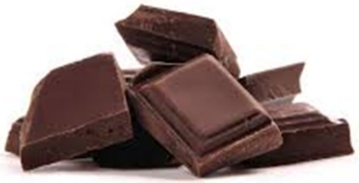

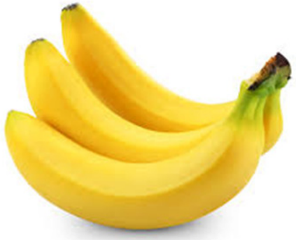

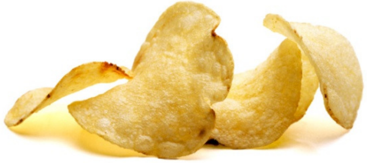

Healthy Start

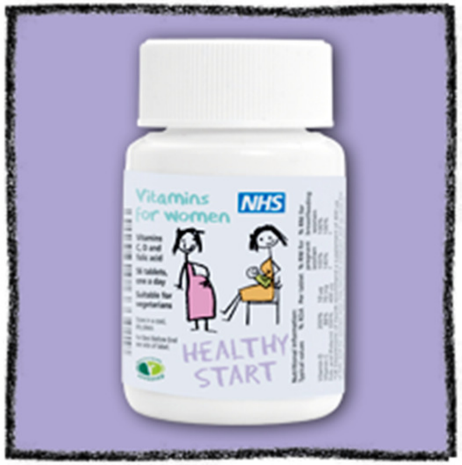

- Vouchers
- Vitamins

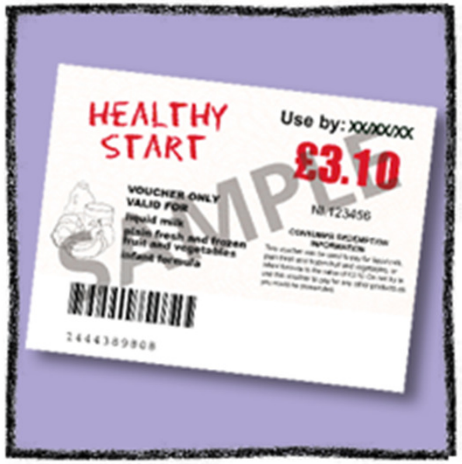

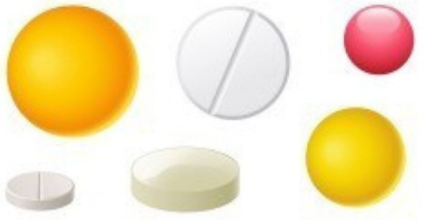

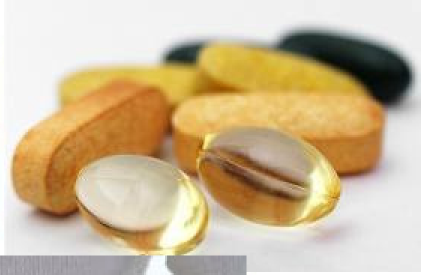

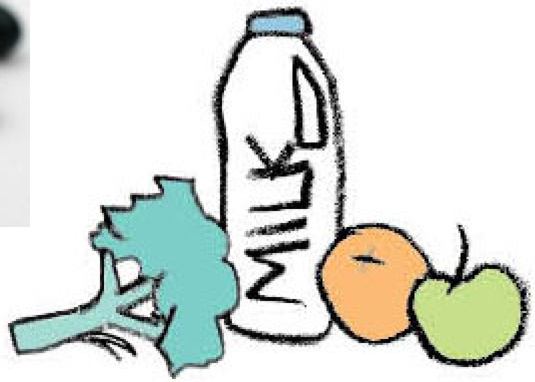

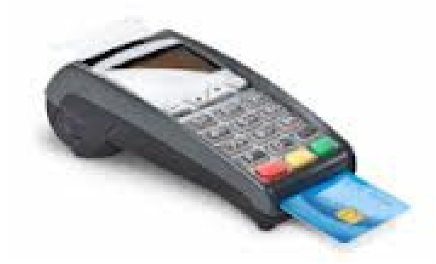

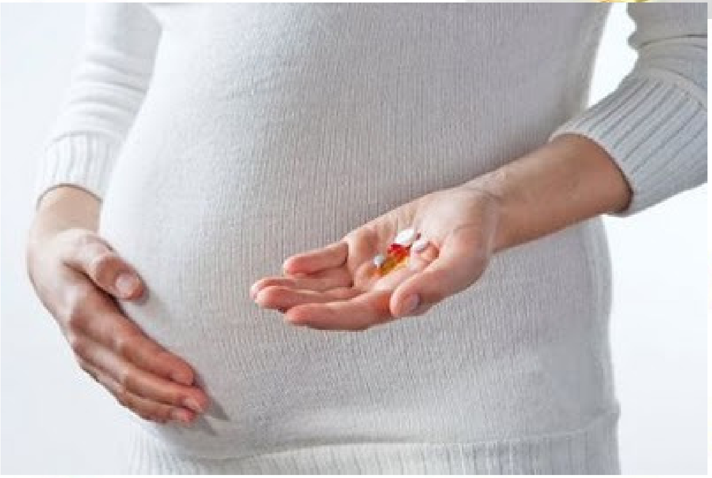

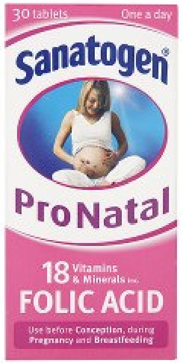

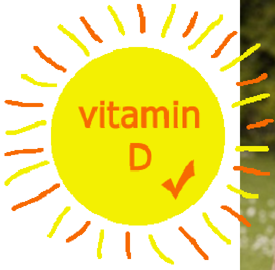

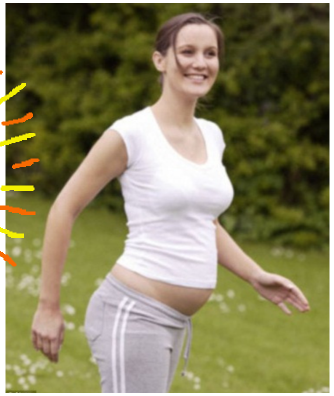

Sources of Information

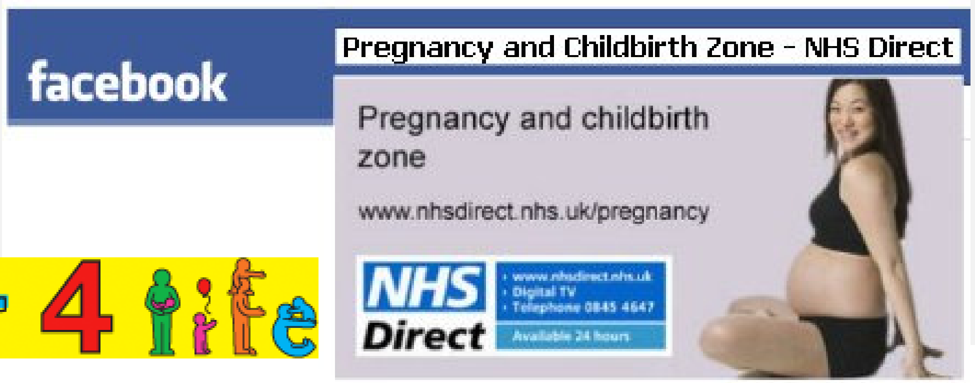

start 4 life

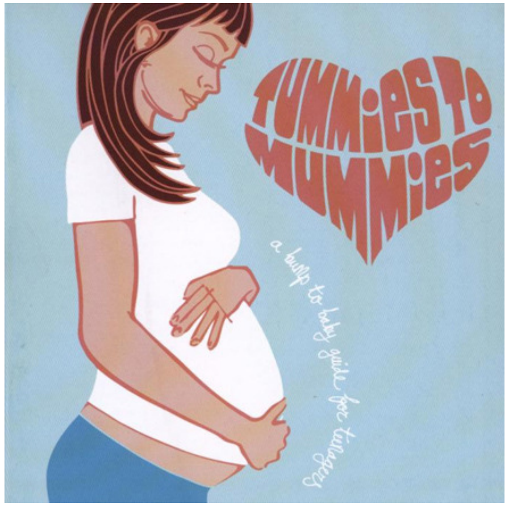

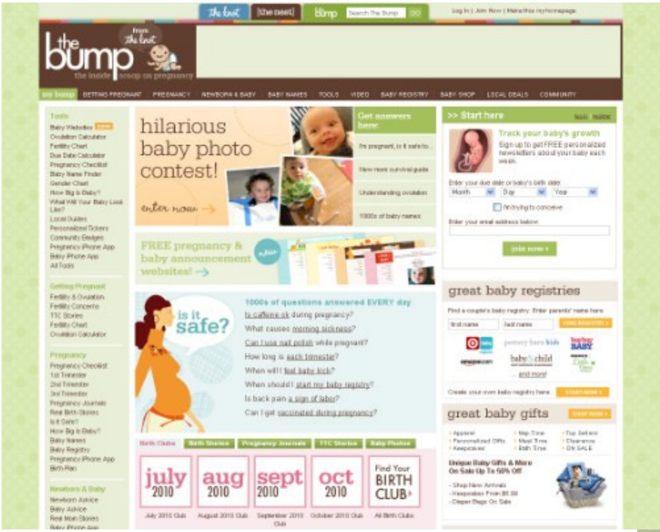

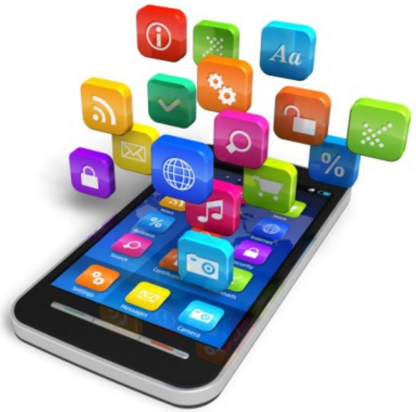

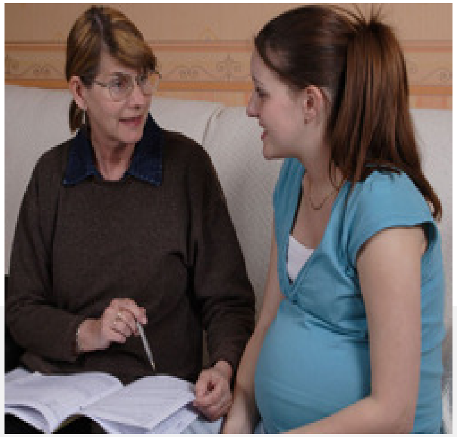

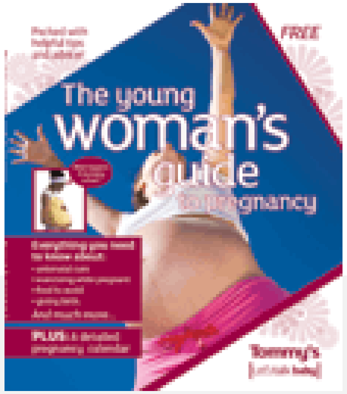

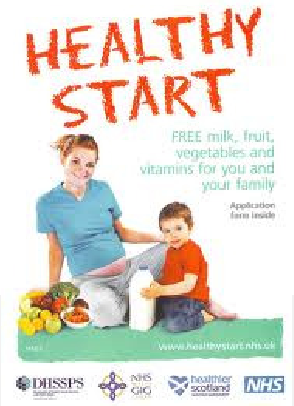

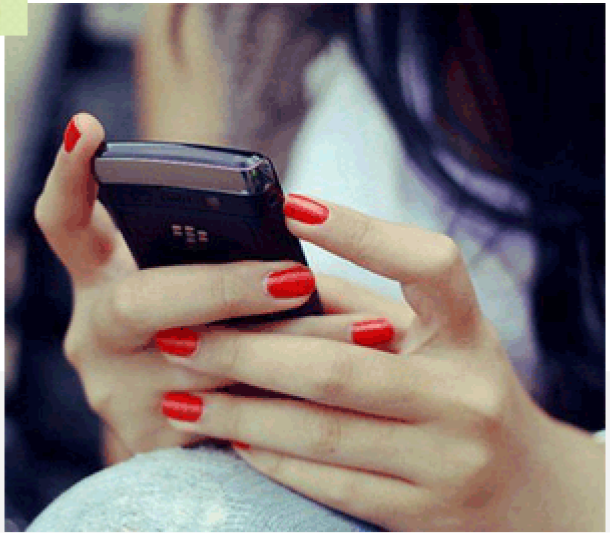

- **If there was one thing you think would make a difference in helping you to make healthier choices what would it be?**

- *Is there anything else you'd like to tell me that's relevant to what we've talked about that we've not covered so far....*

Thank You

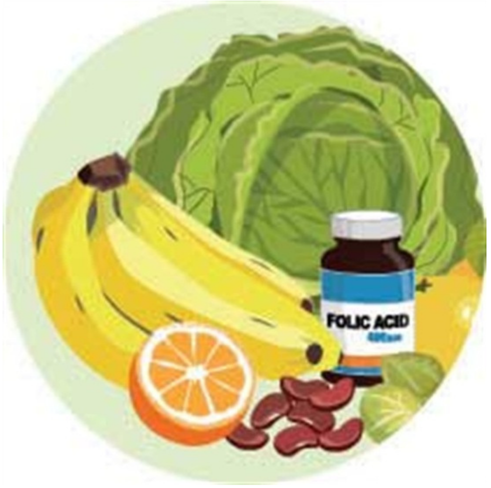

Supplement: Supplementary file 1 — Young Women's Interview Guide. (PDF 1208 kb) [file 40795_2018_254_MOESM1_ESM.pdf]
