# Supplementary material for: Exploring the views of young women and their healthcare professionals on dietary habits and supplementation practices in adolescent pregnancy: a qualitative study
Source: BMC Nutr. 2018 Nov 12;4:45. doi: 10.1186/s40795-018-0254-7 (PMC7050931; doi:10.1186/s40795-018-0254-7)
Supplement: Supplementary file 2 — Table S2. Diet theme, sub-themes and coding with illustrative quotes from young women and health professionals. (DOCX 22 kb) [file 40795_2018_254_MOESM2_ESM.docx]

Additional file 2

**Table S2 Diet theme, sub-themes and coding with illustrative quotes from young women and health professionals**

| *Theme 1* | *Sub-themes* | *Code names* | *Illustrative quotes* |
| --- | --- | --- | --- |
| A time for dietary change amidst confusion | Poor quality habitual diet | Junk food, convenience, take-away | Pop, chips, takeaways, convenience foods, rarely do they have a good diet I find [MIDWIFE]  When they’re hungry just pick up the chocolate bars… they’re just addicted to high sugar diet. [OBSTETRICIAN] |
|  |  | Irregular eating patterns, snacking, grazing | Yeah, I find that they graze a lot. They don't really have like a breakfast, lunch, dinner... they would wake up late, so they miss breakfast… they tend to snack quite a lot. [MIDWIFE]  Mainly crisps; in a day I’d probably have five packets. [YOUNG WOMEN] |
|  |  | Food preference,  resistance | I’m not really good at taking advice any, because I will just eat what I want to eat regardless of what people tell me. [YOUNG WOMEN]  And even when you advise them… they need to eat certain foods, they just say they don’t like them. They don’t like vegetables, they don’t want to eat red meat. [MIDWIFE] |
|  |  | Influence of family, home environment | They grew up in an environment where they don't eat a lot of fresh food then they don't have fresh fruit and vegetables, their patterns are the same. And I've seen that a lot with some clients, their families eat a lot of fast food, the girls eat a lot of fast food [MIDWIFE] |
|  | Social circumstances | Living arrangements, access to storage/cooking facilities | Depends on accommodation doesn’t it up to a large point as to the availability of food, the availability of cooking, utensils. You know, if you haven’t got proper facilities to cook, then things like takeaways, snacking, you know, probably your higher fat less healthy foods really. [FAMILY NURSE PRACTITIONER]  I’ll eat takeout because of where I’m living now. Because we can’t cook, we’re staying in a hostel. [YOUNG WOMEN] |
|  |  | Low income | They would often say I can’t afford to go and buy fresh vegetables and meat all the time [MIDWIFE]  I didn’t have much money so I’d just eat like the cheap stuff, noodles, sandwiches, stuff like that. [YOUNG WOMEN] |
|  |  | Support from others, practical | If they’re living at home or they’ve got a boyfriend who wants to cook for them [MIDWIFE]  Wanting to look after me and make sure I’m eating the fruit. He basically shovels it down me the fruit. [YOUNG WOMEN - talking about her partner]  Yeah, we both cook really. We do it together. [YOUNG WOMEN - cooking with Mum] |
|  | Changes in pregnancy | Healthier choices | I started to eat fruit, like bananas, apples, grapes, and I used to eat broccoli for iron, and more vegetables and more healthy food. [YOUNG WOMEN] |
|  |  | Less healthy choices | I wouldn’t eat that much fatty food before but since I’ve been pregnant I’ve been eating more fatty food. Like I don’t want nothing else, I just want that. [YOUNG WOMEN] |
|  |  | Healthy additions | I guess it’s junk food. That’s not really healthy. Different types like burgers, chips, kebabs. Yeah, but I’ve tried to balance it out in my pregnancy, I tried to get a few fruits in there. [YOUNG WOMEN] |
|  |  | Cravings, sickness, nausea | At the minute I’m craving mash, mash or veg. [YOUNG WOMEN]  I used to eat a lot of veg, but now it makes me want to be sick. [YOUNG WOMEN] |
|  |  | Food avoidance | Now I don’t know what soft cheese I can eat and what soft cheese I can't eat, so I just avoid the whole of it. [YOUNG WOMEN]  So they’re cutting out a lot of things in their diet that they can have that are good for them because they think and they’ve heard, they’ve got the wrong information. [DFAMILY NURSE PRACTITIONER2] |
|  | Motivation to change | Making the connection (+ve) | I think the motivation I get to eat veg and fruit, because I was a stubborn girl, I wouldn’t eat it, was my baby, because as soon as I found out I was pregnant I was like, right, this is where I’m going to have to change, this is where I need to start eating good stuff to make my baby be healthy because if I didn’t do that then God knows what the baby would be like when it gets older, so no, that was my motivation straightaway. [YOUNG WOMEN]  I decided to eat a lot more fruit and veg, just because I thought the baby’s still getting everything they need, and then it’s healthy for me I’m not going to be like putting loads of weight on. [YOUNG WOMEN]  So baby just likes it, it makes me feel better in my body every day and obviously I’ve got a baby inside, so I have to look after it. [YOUNG WOMEN] |
|  | Barriers to change | Cooking skills and confidence | I know I have to eat and I have to make myself something to eat, so I have to do it. I’m not a big cooker. I haven’t been a big cooker since I've been pregnant. [YOUNG WOMEN]  I can't be bothered to cook most of the time, and it’s easier just to have snacks. [YOUNG WOMEN]  They don’t really have skill of cooking or preparing a meal [FAMILY NURSE PRACTITIONER] |
|  |  | Myths and misconceptions | A smaller baby is going to be easier to get out. A smaller baby means you're not going to tear. What they don't realise is a smaller baby means that the baby might not be able to cope… and might get very distressed. [MIDWIFE]  I think it’s more to do with labour. I don’t want to make my baby overgrown. [YOUNG WOMEN - talking about not wanting to eat]  So you have to put those things right, because all they think is "tiny baby, easy labour". [MIDWIFE] |
|  | Healthy Start vouchers for food | Supporting healthy additions | I've been getting fruit, just fruit. Literally I've been getting grapes and oranges, apples, bananas, pineapple, melon. [YOUNG WOMEN]  I think my mum will use them for the things that she’s buying in for me like for fruit and vegetables [YOUNG WOMEN] |
|  |  | Mis-use of vouchers | We know they’re misused, sadly… we’ve a lot of work for us to be doing around naming and shaming these small corner shops that give cigarettes out and alcohol [FN]  The shop up road that lets me get bread. So if I've run out of bread or stuff like, I can get bread and that on it [YOUNG WOMEN] |
