# Supplementary material for: Exploring the views of young women and their healthcare professionals on dietary habits and supplementation practices in adolescent pregnancy: a qualitative study
Source: BMC Nutr. 2018 Nov 12;4:45. doi: 10.1186/s40795-018-0254-7 (PMC7050931; doi:10.1186/s40795-018-0254-7)
Supplement: Supplementary file 3 — Table S3. Supplementation theme, sub-themes and coding with illustrative quotes from young women and health professionals. (DOCX 20 kb) [file 40795_2018_254_MOESM3_ESM.docx]

Additional file 3

**Table S3. Supplementation theme, sub-themes and coding with illustrative quotes from young women and health professionals**

| *Theme 2* | *Sub-themes* | *Code names* | *Illustrative quotes* |
| --- | --- | --- | --- |
| Erratic adherence to supplementation despite uncertainty | Initiation of supplements | Crucial first contact | I think if we gave them then they would take them. I think if we sort of were emphasising the importance more, then obviously I think they would comply better. [MIDWIFE]  I don’t understand why at booking, you know, why the midwife can’t just give them their vitamins. [FAMILY NURSE PRACTITIONER] |
|  |  | Prompted by partner, family & friends | My mum said like as soon as I found out I was pregnant she was like you need to take some vitamins, because obviously it helps to get the folic acid there straight away. [YOUNG WOMEN]  My mum’s mate was pregnant at the same time and she said oh are you taking this? [YOUNG WOMEN] |
|  |  | Late presentation and delayed start | So they sometimes don’t take folic acid at all. Some of them don’t even have their pregnancies diagnosed until they’re 16 weeks or more [MIDWIFE]  I started taking it properly from 13 weeks onwards, because we didn’t know if I was going to be keeping the baby or not. [YOUNG WOMEN] |
|  |  | Proactive approach - identifying deficiencies | But one of the things that I would like us to be much more circumspect about is monitoring their haemoglobin levels, more frequently than older women in pregnancy, because when they drop their haemoglobin they can drop it quite precipitously [OBSTETRICIAN] |
|  | Varied knowledge & awareness | Lack of awareness | I didn’t even know anyone took vitamins when they were pregnant I thought it just happened and you just got on with it and you were all right. [YOUNG WOMEN] |
|  |  | Folic acid | And do you know why you need Folic acid? [Interviewer] It helps your baby’s bone, its bones develop or something. [YOUNG WOMEN] |
|  |  | Iron | I know that iron is something about brain development with the baby. [YOUNG WOMEN] |
|  |  | Vitamin D | Vitamin D, most people still haven’t heard about that. Every time I talk about it people are surprised. [MIDWIFE] |
|  |  | Healthy Start vitamins | I think there’s vitamin C, D and E…I think something about the baby’s bones….I can’t remember the rest. I just can’t remember. [YOUNG WOMEN - when asked about content] |
|  |  | Multi-vitamins | For some of the teenagers because they’ve got such bad diets… it might be worth trying a pregnancy multivitamin, because that’s just got a little bit of everything in it [MIDWIFE] |
|  | Barriers to use, irregular use | Forgetting, other priorities | Sometimes I forget, but normally I take it every morning but if I forget in the morning I remember in the night time. [YOUNG WOMEN]  A lot of people I see their lives are quite chaotic so remembering to take a tablet is probably not at the top of their list really [MIDWIFE]  I do remember to take my folic acid, but sometimes like in a day just forget to take it. [YOUNG WOMEN] |
|  |  | Stopping early, concerns about safety | 12 weeks… you can’t have it after that, you have to stop because it’s not meant to be good for you [YOUNG WOMEN - talking about folic acid]  Avoid all medication, and they’ve seen the supplements as medication [MIDWIFE] |
|  |  | Lack of personal relevance | I do see the need for them if necessary… I’m not sick, it’s different if there was any problems with baby, her scan was perfect, and everything came back fine, I don’t have any worries, even her Down Syndrome thing came, everything is just going well with how I've been… natural [YOUNG WOMEN - didn't take any supplements during pregnancy] |
|  |  | Tablet format, sickness | No, I don’t do tablets so I’ve not taken anything at all. [MIDWIFE]  Because like when you’re pregnant you have like, like your folic and a fizzy drink you feel kind of sick. [YOUNG WOMEN] |
|  | Increasing adherence | Acceptance, best for baby | I know I'm meant to take it but I don’t know why. I just take it because I've been told I need to. [YOUNG WOMEN] |
|  |  | Incentivising with outcomes, routine tests | If you say to them you need to bring your iron up if you want to have a nice normal labour, with just the midwives on the birth centre rather than on the medical side of the hospital… then they're like oh, okay, I need to do this. [MIDWIFE]  If I take your bloods in four weeks and it's not come up any, you haven’t been taking the tablets and I'll know you haven’t been eating well. [MIDWIFE] |
|  |  | Planning prompts, cues | I used to forget to take them but then I started putting like the little package in my purse, because I use that every day. So I’d see it, I’d remember to take it. [YOUNG WOMEN]  Well, what it is they’re right next to the kettle in the morning and I have a brew first thing in the morning like when I get up any, and they’re just right there. [YOUNG WOMEN] |
|  |  | Relationships and reminders | It’s about them having a good relationship with the midwife who is going to remind them, about the family and friends reminding them. I think once we’ve got over the barriers then I think the girls will take them, but it’s very much dependent on the support they’ve got and being reminded by the midwife. [MIDWIFE] |
|  |  | Pragmatic approach | If you’ve missed a couple of days, fine, don’t panic, but carry on. I don’t make them think that it’s got to be all on, it’s preferably every day but we know that that’s not always practical and if we’re realistic, they won't. [MIDWIFE] |
|  |  | Tablet size, smaller pills | I can take these because they’re not that big [health start vitamins], and obviously and if they are like kind of big and fat [multi-vitamins] I just throw it up. [YOUNG WOMEN]  It just goes down straightaway. You don’t even know that you took a tablet. [YOUNG WOMEN] |
|  |  | Alternative supplementation format | It wouldn’t be so hard to make a smoothie with folic acid in it [YOUNG WOMEN]  That would be the best thing ever for pregnancy, chocolate that is good for you. Chocolate with all your vitamins in, every woman would eat that every day [YOUNG WOMEN] |
