# Supplementary material for: Exploring the views of young women and their healthcare professionals on dietary habits and supplementation practices in adolescent pregnancy: a qualitative study
Source: BMC Nutr. 2018 Nov 12;4:45. doi: 10.1186/s40795-018-0254-7 (PMC7050931; doi:10.1186/s40795-018-0254-7)
Supplement: Supplementary file 4 — Table S4. Information and support theme, sub-themes and coding with illustrative quotes from young women and health professionals. (DOCX 20 kb) [file 40795_2018_254_MOESM4_ESM.docx]

Additional file 4

**Table S4. Information and support theme, sub-themes and coding with illustrative quotes from young women and health professionals**

| *Theme 3* | *Sub-themes* | *Code names* | *Illustrative quotes* |
| --- | --- | --- | --- |
| Seeking relevant and reliable information and support | Promoting healthy pregnancy | HP's role, opportunity, education | I think sometimes when it’s coming from a professional, certainly if you’ve got a good relationship, they do take it on board - some of the girls any [MIDWIFE]  I think the majority of our role is to do with having a healthy mum and a healthy baby, that's what you want at the outcome, so absolutely diet is part of that [MIDWIFE]  It's that window of opportunity to educate someone to make better changes for themselves and for their future. [MIDWIFE] |
|  |  | Family nurse practitioner approach | Before we even touch on diet, we’d actually try and tap into that intrinsic motivation about the baby… on our first visit we look at building the baby’s brain and directly linking it to eating healthier foods. [FAMILY NURSE PRACTITIONER] |
|  |  | Obstetrician role | But I don’t routinely talk about the sort of stuff that they should be eating, they should have been given the literature and I would expect conversations to have been had surrounding the literature that they’ve received. [OBSTETRICIAN] |
|  |  | Supporting change, making the connection | So it's us helping them to understand that just because you can't see the baby, and just because you don't know what's going on in there, it doesn’t mean that what you're eating is not having an effect [MIDWIFE] |
|  |  | Additional support, skills | We have tried to set up a Good Grub Club where they will come and they’ll cook a meal with vegetables… the attendance for that was very poor. [MIDWIFE] |
|  | Barriers to information provision, tailored support | Time constraints & conflicting demands | Don’t have as much time to talk about diet as possibly we’d like to, which is why we use these leaflets and hope that they will read them [MIDWIFE].  But we don’t really talk about much, she just checks me, we don’t talk about much. She checks me, asks me if I have any questions, which I usually don’t. [YOUNG WOMEN - talking about routine antenatal visits with her [MIDWIFE] |
|  |  | Conflicting advice, consistency | I think that’s a big barrier, that it’s something new and why are you taking those pregnancy vitamins when the mum didn’t take them or the older sister didn’t take them [YOUNG WOMEN]  That you shouldn’t take the vitamins, because it may cause a miscarriage - I've come across that twice. Very challenging. [MIDWIFE] |
|  |  | Negative messages, directive approach | She said that I shouldn’t eat prawns and I shouldn’t eat tuna and I shouldn’t eat red meat, I shouldn’t eat this I shouldn’t eat that. [MIDWIFE]  YOUNG WOMEN: I did read, I read everything that I was given but I don’t really remember any of it. I: Did your midwife go through the leaflets…? YOUNG WOMEN: She just gave it to me, she didn’t go through it”. [YOUNG WOMEN] |
|  |  | Understanding social circumstances, context of young women's lives | If you just give people advice and don’t look at what else is going on in their lives and where they’re coming from and understanding about them my experience is you don’t get very far. [FAMILY NURSE PRACTITIONER]  Yeah, we see them a lot at home rather than in clinics, because of the nature of their situation. We want to see their living environment… And you can see what they've got available to them with regards to cooking and things. [MIDWIFE] |
|  | Written resources | Written resources - value | I find the physical books much easier... whereas the Internet you’ve got to be searching and you don’t know if you’re finding the right information, so I think booklets are easier. [YOUNG WOMEN] |
|  |  | Written resources - apathy | Well I got leaflets but I didn’t read them because to be honest with you leaflets that you get these days are so packed full of information, very tiny writing, you just can’t be bothered. [YOUNG WOMEN]  It’s finding the right thing for young people, because they don’t like anything too heavy or too wordy [MIDWIFE] |
|  | Digital technologies, alternative formats, accessing information & support | Online resources - video format | I do watch online videos on YouTube of other young mums, so I type in 33 weeks pregnancy updates and the women, people usually say what kind of tablets, vitamin’s they’re into, what veg and fruits, things they eat. [YOUNG WOMEN]  We look for things on YouTube, anything that's going to be short and quick, entertaining, that's going to give them the right information. [MIDWIFE] |
|  |  | Online resources - social media (YOUNG WOMEN) | I go on NHS I know all right, these are doctors and stuff, so it’s more trustworthy. Whereas I go on Facebook more... so if I know it’s still the same NHS people that are providing this information, but they’re providing it on Facebook, you’ll find me more on Facebook. Especially if it’s young mums, I think you’d find a lot more on them on social networking. [YOUNG WOMEN] |
|  |  | Web based, relevant content tailored resources | Just the NHS websites really. I know there’s things like BabyCenter and Mumsnet but I don’t think they’re really young parents orientated. And I don’t really know of any others to be honest. I haven’t looked for them myself so I wouldn’t know [MIDWIFE]  Don’t actually have to have cartoons and sound like you’re talking to a five year old, which is quite annoying with a lot of the videos, you don’t need to speak like [we] don’t understand, and we’re not children. [YOUNG WOMEN] |
